# Functional characterization of a novel protein-coding circular RNA, circRNA_1193, from the *mAAP* gene in silkworm and its role in antiviral defense against BmCPV

**DOI:** 10.1128/mbio.00125-25

**Published:** 2025-03-28

**Authors:** Song Li, Zeen Shen, Hongchuan Zhao, Xialing Chen, Qunnan Qiu, Xinyu Tong, Min Zhu, Xing Zhang, Chengliang Gong, Xiaolong Hu

**Affiliations:** 1 School of Life Sciences, Soochow University 12582 , Suzhou, China; 2 School of Chemistry and Life Sciences, Suzhou University of Science and Technology 66339 , Suzhou, Jiangsu, China; University of Würzburg, Würzburg, Germany; Helmholtz-Institute for RNA-based Infection Research, Würzburg, Germany

**Keywords:** silkworm, BmCPV, circRNA_1193, ORF, virus replication

## Abstract

A novel circular RNA, circRNA_1193, which originates from the *membrane alanyl aminopeptidase-like* gene in silkworms, was explored for its potential function and regulatory mechanism. We validated the presence of circRNA_1193 in *Bombyx mori* cytoplasmic polyhedrosis virus (BmCPV)-infected silkworm ovary cell line (BmN) cells through a combination of reverse transcription polymerase chain reaction, Northern blotting, and *in situ* hybridization. CircRNA_1193 exhibited tissue-specific expression, being highly enriched in the midgut and Malpighian tubules, and displayed a specific response to BmCPV infection, but not to *Bombyx mori* nucleopolyhedrovirus or lipopolysaccharide. Functional analyses revealed that the overexpression of circRNA_1193 suppressed BmCPV replication, whereas its knockdown increased viral replication. Bioinformatic analyses revealed potential internal ribosome entry sites, m6A methylation sites, and open reading frames (ORFs) within circRNA_1193, suggesting its potential coding capacity. We confirmed the translation of the ORF by constructing a DsRed reporter vector and demonstrating DsRed expression in transfected cells. Furthermore, a mutation of the start codon within circRNA_1193 abolished its antiviral activity, highlighting the crucial role of the translated protein, which is 35 kDa and is designated as VSP35. Furthermore, our data suggest that the formation of circRNA_1193 relies on reverse complementary flanking sequences. These findings unveil a novel protein-coding circular RNA in silkworms that plays a critical role in antiviral defense.

**IMPORTANCE:**

This study identified a novel circular RNA, circRNA_1193, in the silkworm *Bombyx mori*, and revealed its critical role in antiviral defense against *Bombyx mori* cytoplasmic polyhedrosis virus (BmCPV). We demonstrated that circRNA_1193 exhibits tissue-specific expression, is upregulated in response to BmCPV infection, and possesses antiviral activity. Importantly, we show that circRNA_1193 encodes the viral protein VSP35, which is essential for its antiviral function. These findings provide new insights into the complex regulatory mechanisms of circular RNAs in antiviral immunity and underscore the potential of circular RNAs as therapeutic targets in viral diseases. The identification of a protein-coding circular RNA with antiviral activity in *B. mori* has broader implications for understanding the evolution and diversity of host defense mechanisms against viruses.

## INTRODUCTION

Circular RNAs (circRNAs) are a class of noncoding RNAs characterized by their covalently closed structure, which lacks both a 5´ cap and a 3´ polyadenylation tail. These unique properties allow circRNAs to engage in various biological functions, including acting as molecular sponges for microRNAs (miRNAs), regulating transcription, and, more recently, being recognized for their potential coding capabilities (1, 2). Emerging evidence suggests that circRNAs play pivotal roles in diverse cellular processes and are implicated in the response to viral infections (3–5). Unlike traditional linear RNAs, circRNAs possess a stable structure that makes them resistant to degradation by exonucleases, which may confer advantages in terms of gene regulation and cellular stability (6, 7). Recent studies have shown that certain circRNAs can encode proteins, expanding our understanding of their functional repertoire beyond the traditional view of noncoding RNAs (8, 9).

In the context of viral infections, circRNAs have been implicated in various pathways that enhance or inhibit viral replication. For example, some circRNAs reportedly act as sponges for specific miRNAs, thereby regulating the expression of host factors that may facilitate or impede the viral life cycle (10, 11). Additionally, circRNAs can engage with viral proteins, potentially influencing viral pathogenesis and host immune responses (12, 13).

In silkworms, the investigation of circRNAs is particularly relevant given their use in sericulture and as model organisms for studying insect physiology and pathology. Understanding the interactions between circRNAs and viruses such as *Bombyx mori* cytoplasmic polyhedrosis virus (BmCPV) could provide insights into the innate immune responses of silkworms, as well as potential avenues for enhancing disease resistance (14, 15). In our previous study, we mapped the global expression patterns of circRNAs in the midgut of silkworms following BmCPV infection. Our findings revealed that a significant number of circRNAs were dysregulated due to the viral infection, with functional predictions suggesting that most of them are linked to metabolism and innate immunity (14). Notably, many of these circRNAs were reported for the first time in silkworms, highlighting their potential regulatory roles in the pathogenesis associated with BmCPV infection. Additionally, we discovered that the circRNA circEgg, formed by the splicing of the 9th to 13th exons of the *B. mori* histone-lysine N-methyltransferase eggless (BmEgg) gene, plays a role in regulating histone modification by sponging bmo-miR-3391-5p and encoding the circEgg-P122 protein (16). Here, one circRNA of particular interest is circRNA_1193, which is predicted to be derived from exons 5 to 9 of the *B. mori membrane alanyl aminopeptidase-like* (*mAAP*) gene. Previous studies have indicated that the *mAAP* gene is responsive to BmCPV infection, a significant pathogen affecting silkworm larvae (17). Furthermore, membrane alanyl aminopeptidase N was identified as a receptor for pea enation mosaic virus (18). However, the hidden mechanisms through which circRNA_1193 impacts viral replication and its functional significance remain largely undefined.

In this study, to explore the hidden regulatory roles of circRNA_1193 in the context of viral infection, we elucidated the authenticity, expression patterns, and functional roles of circRNA_1193 in the context of BmCPV infection. By investigating the potential coding capacity of circRNA_1193 and its effects on viral replication, we hope to contribute to the growing body of knowledge on circRNAs and their regulatory functions in viral pathogenesis.

## RESULTS

### The dynamic landscape of the silkworm circRNAs during BmCPV infection

To investigate the dynamic landscape of circRNA expression in silkworms during BmCPV infection, we analyzed the expression patterns of 20 specific circRNAs in BmN cells infected with BmCPV at 24, 48, 72, and 96 h post-infection. Total RNA was treated with RNase R to remove linear RNAs and enrich for circular RNAs. Reverse transcription polymerase chain reaction (RT-PCR) was conducted via divergent primers to amplify 20 silkworm circRNAs (circRNA_0325, _1029, _1193, _1468, _1750, _2200, _3092, _3104, _3184, _3285, _3827, _4088, _4499, _4541, _4943, _5322, _5354, _5663, _5664, _6595), which were selected from our previous high-throughput sequencing data because of their high expression levels induced by BmCPV.

**Fig 1:**
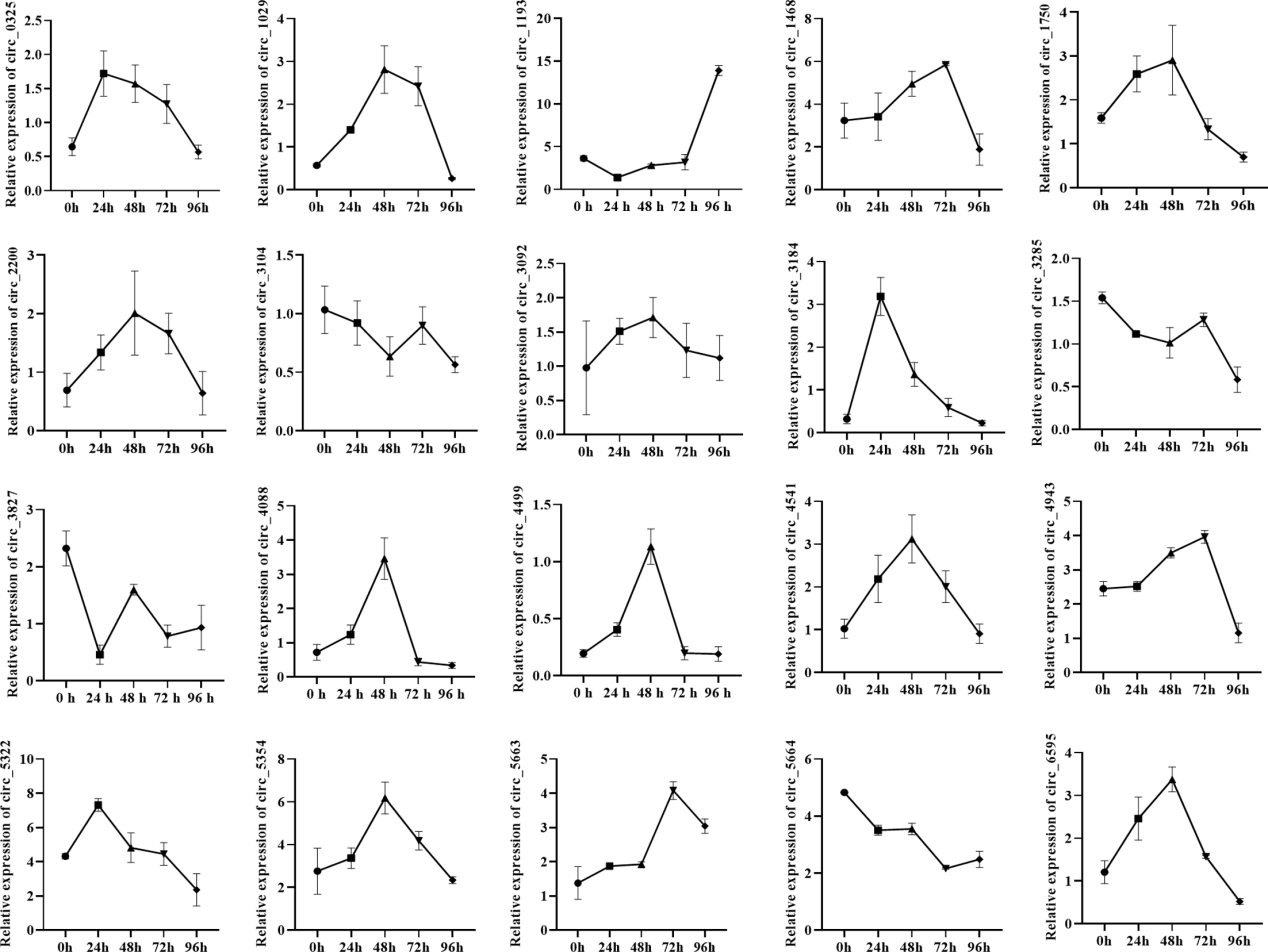
Dynamic landscape of silkworm circRNA expression during BmCPV infection. Twenty silkworm circRNAs (circRNA_0325, _1029, _1193, _1468, _1750, _2200, _3092, _3104, _3184, _3285, _3827, _4088, _4499, _4541, _4943, _5322, _5354, _5663, _5664, _6595) were selected from our previous high-through sequencing data. Total RNA was extracted from BmN cells and reverse transcribed into cDNA. Real-time PCR was performed via divergent primers targeting the selected circRNAs, with *RP49* serving as the reference gene.

Our analysis revealed significant fluctuations in the expression levels of these circRNAs across the different time points (Fig. 1). An initial baseline expression profile was observed at 24 h post-infection. As the infection progressed to 48 and 72 h, many circRNAs exhibited significant upregulation or downregulation, suggesting their involvement in the cellular response to viral infection. At 96 h post-infection, the expression patterns continued to evolve, indicating that a complex regulatory landscape mediated the response of the silkworm to BmCPV. Among them, circRNA_1193 was highly induced and expressed following BmCPV.

These findings highlight the dynamic nature of circRNA expression in silkworms during BmCPV infection, suggesting that these molecules may play crucial roles in the host immune response and metabolic adjustments in combating viral challenges.

### Experimental confirmation of silkworm circRNA_1193 derived from BmCPV-infected cells

**Fig 2:**
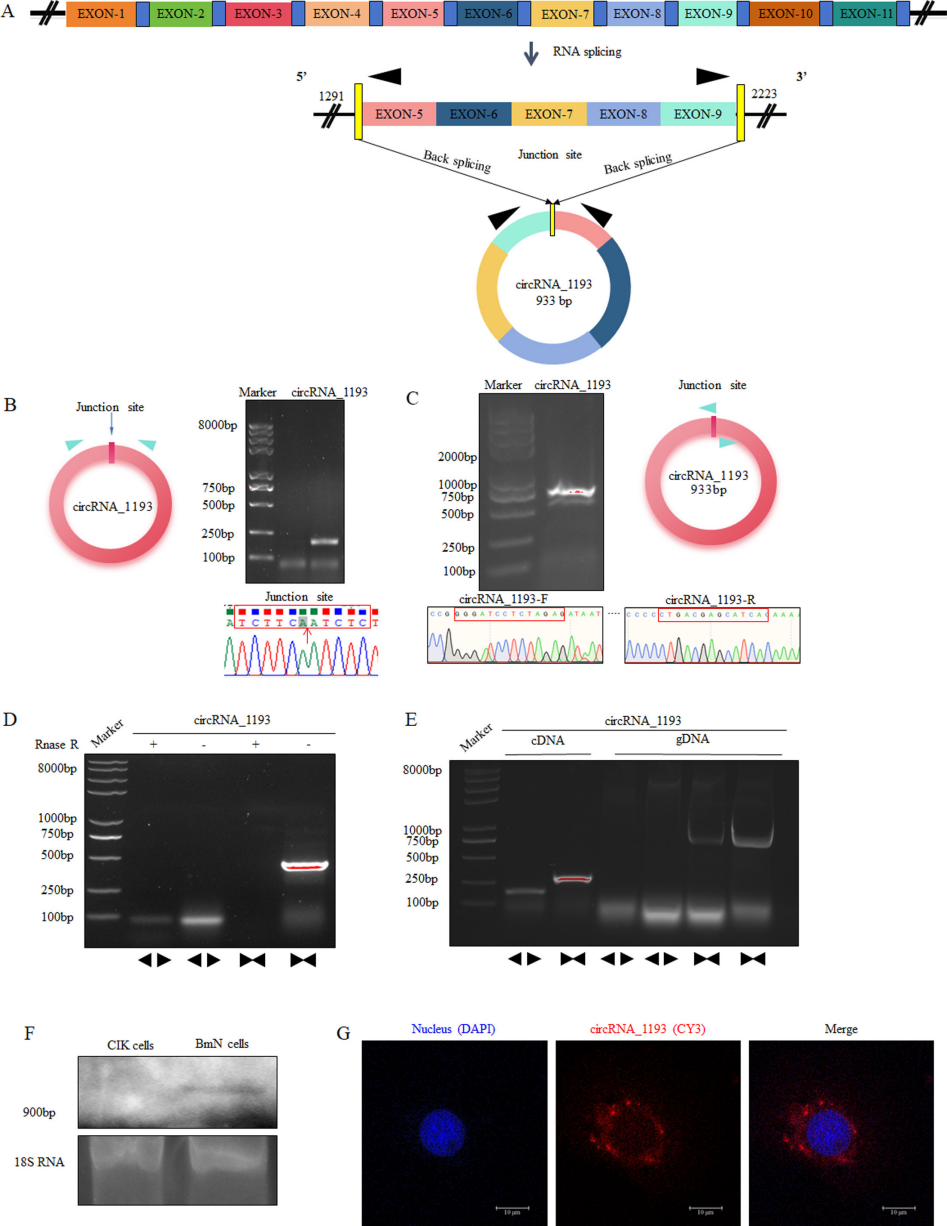
Experimental confirmation of silkworm circRNA_1193 derived from BmCPV-infected cells. (A) Genomic locus of silkworm from which circRNA_1193 is derived. (B) Detection of the junction site of circRNA_1193 via divergent primers. Total RNA extracted from BmN cells was amplified via RT-PCR using divergent primers targeting the junction site. The PCR products were then analyzed via electrophoresis and Sanger sequencing. The red arrow indicates the junction site. (C) Detection of the complete sequence of circRNA_1193 via convergent primers. Total RNA extracted from BmN cells was amplified via RT-PCR using convergent primers. The PCR products were then analyzed via electrophoresis and Sanger sequencing. (D) Confirmation of circRNA_1193 as a ring molecule. Linear RNA was removed from total RNA extracted from BmN cells via digestion with RNase R. Convergent and divergent primers were used to amplify the complete sequence of circRNA_1193 and the junction site. (E) Confirmation of circRNA_1193 as a covalently closed ring structure. Total RNA extracted from BmN cells was reverse transcribed into cDNA. gDNA extracted from BmN cells was used as a template for amplifying the junction site of circRNA_1193. The amplified products were detected via electrophoresis. (F) Northern blotting validation of circRNA_1193. Total RNA was extracted from 1 × 10^5^ BmN cells for Northern blotting via a biotin-labeled probe. CIK represents grass carp kidney cells as a control, and BmN represents silkworm ovary cells. (G) *In situ* hybridization assay of circRNA_1193. A total of 1 × 10^5^ BmN cells were cultured in 24-well plates for 24 h. The cells were hybridized *in situ* with an oligonucleotide biotin probe designed according to the circRNA_1193 junction site. The secondary antibody was a Cy3-labeled streptavidin (red fluorescence) antibody. Nuclei were stained with 4′,6-diamidino-2-phenylindole (DAPI) (blue fluorescence).

Bioinformatic analysis revealed that circRNA_1193 (933 nt) originates from exons 5 to 9 of the silkworm *mAAP* gene (Fig. 2A). To validate the existence of circRNA_1193, we employed a series of molecular biology techniques. We performed RT-PCR on RNA samples treated with RNase R, an enzyme that selectively degrades linear RNAs and is enriched with circular RNAs. RT-PCR was performed using both divergent and convergent primers. Divergent primers were designed to target the unique junction site present in circRNA_1193 (Fig. 2B), whereas convergent primers served as a complementary approach to verify the presence of circRNA_1193 through traditional linear amplification. Sanger sequencing confirmed the identity of the amplified product (Fig. 2C). To further confirm the circular structure of circRNA_1193, we performed PCR amplification via complementary DNA (cDNA) and genomic DNA (gDNA) extracted from BmN cells, which targeted the complete sequence of circRNA_1193 or its junction site (Fig. 2D and E). The sequencing results validated the circular form of circRNA_1193 and confirmed its origin from the predicted genomic locus.

In addition to RT-PCR and Sanger sequencing, we further validated the presence and expression of circRNA_1193 via Northern blotting and *in situ* hybridization. For Northern blotting, total RNA extracted from BmCPV-infected silkworm cells was separated by size on an agarose gel, transferred to a PVDF membrane, and hybridized with a biotin-labeled probe specific for the unique junction site of circRNA_1193. The results confirmed the presence of circRNA_1193, as indicated by a distinct band corresponding to the expected size (Fig. 2F). This band was absent in RNA samples extracted from *Ctenopharyngodon idellus* kidney cells (CIK cells), confirming the specificity of the signal to circRNA_1193. *In situ* hybridization revealed a distinct signal localized within the cytoplasm of BmN cells, demonstrating the active transcription of circRNA_1193 (Fig. 2G).

Collectively, our experimental findings confirmed the presence of circRNA_1193 in BmCPV-infected silkworm cells. These results provide valuable insights into the potential role of circRNA_1193 in BmCPV infection and highlight the importance of circRNAs in the silkworm response to viral challenge.

### The expression of circRNA_1193 was significantly elevated in response to BmCPV infection

**Fig 3:**
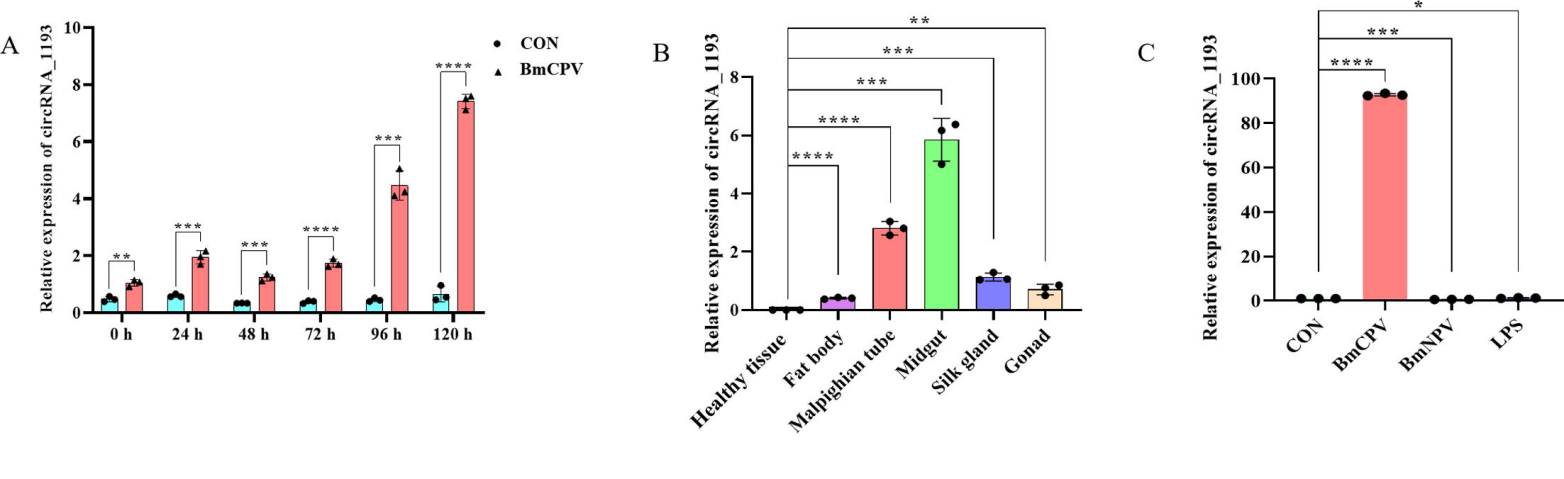
Expression of circRNA_1193 in response to viral infection and immune stimuli. (A) Expression of circRNA_1193 in different silkworm tissues. Different silkworm tissues were dissected for total RNA extraction. The expression of circRNA_1193 was detected via real-time PCR, with *RP49* used as the reference gene. (B) Expression of circRNA_1193 in BmCPV-infected BmN cells. Real-time PCR was performed to detect the expression level of circRNA_1193 via divergent primers. Total RNA was extracted from normal BmN cells and BmCPV-infected BmN cells, and reverse transcribed into cDNA via random primers. *RP49* served as the reference gene. (C) Immune response of circRNA_1193 to BmCPV, BmNPV, or LPS in BmN cells. BmN cells were infected with BmCPV, BmNPV, and LPS. Total RNA was extracted from the cells, and the expression of circRNA_1193 was detected via real-time PCR, with *RP49* used as the reference gene.

To investigate the potential role of circRNA_1193 in the silkworm response to BmCPV infection, we examined its expression pattern. CircRNA_1193 was highly expressed in both the midgut and Malpighian tubules of silkworms (Fig. 3A), suggesting that a targeted response is potentially linked to metabolic and immune pathways active during BmCPV infection. Furthermore, the relative expression level of circRNA_1193 gradually increased over time, reaching its highest value at 120 h post-infection (Fig. 3B). Exposure of BmN cells to BmCPV, *Bombyx mori* nucleopolyhedrovirus (BmNPV), or lipopolysaccharide (LPS) resulted in a distinct increase in circRNA_1193 levels specifically following BmCPV exposure (Fig. 3C), suggesting a potential role in the silkworm’s defense mechanisms against this virus. Notably, circRNA_1193 did not exhibit a significant expression change in expression in response to BmNPV infection or LPS treatment, suggesting a specific response to BmCPV and a potential lack of involvement in broader immune responses activated by other viral infections or gram-negative bacterial challenge. These findings collectively suggest that circRNA_1193 plays a specialized role in the silkworm response to BmCPV. Its high expression in critical tissues involved in immunity and metabolism, coupled with its selective responsiveness to BmCPV, points to its potential role as a key player in the host-pathogen interaction.

### Functions of circRNA_1193 during viral infection

**Fig 4:**
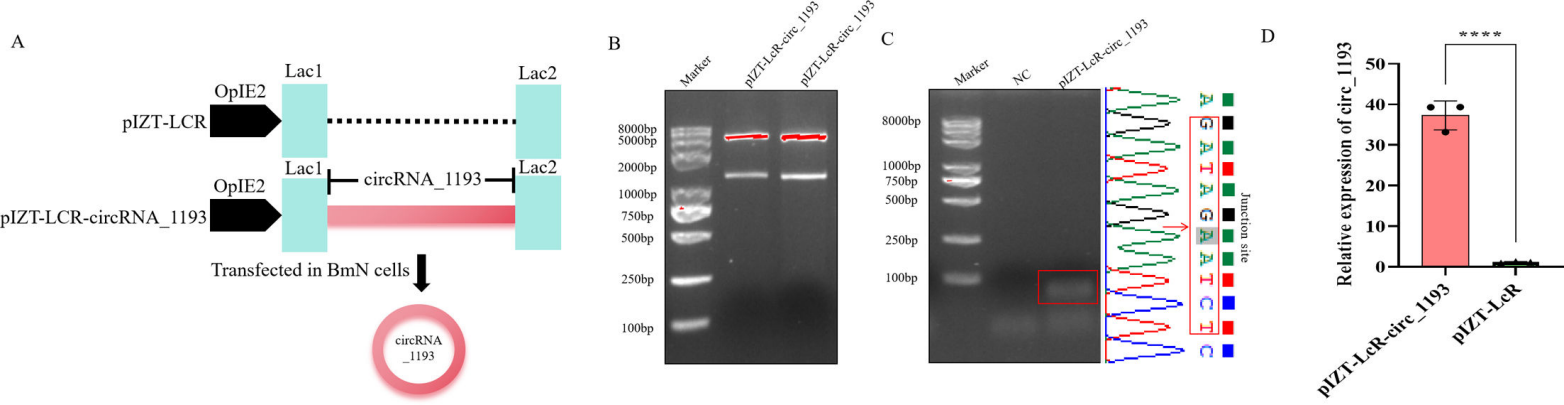
Construction and validation of the circRNA_1193 expression vector pIZT-LcR-circRNA_1193. (A) Schematic representation of the pIZT-LcR-circRNA_1193 transient expression vector construction. (B) Restriction enzyme digestion of pIZT-LcR-circRNA_1193. The vector was digested with *Bam*H I and *Eco*R I, and the resulting fragments were analyzed via electrophoresis. (C) Validation of circRNA_1193 formation in pIZT-LcR-circRNA_1193-transfected cells. Total RNA was extracted from the pIZT-LcR-circRNA_1193-transfected cells and reverse transcribed into cDNA via random primers. RT-PCR was performed to detect the junction site of circRNA_1193, and the amplified products were analyzed via electrophoresis. Sanger sequencing was used to confirm the junction site and flanking sequences of circRNA_1193. The red arrow indicates the junction site. (D) Real-time PCR analysis of circRNA_1193 expression in pIZT-LcR-circRNA_1193-transfected BmN cells. Real-time PCR was performed via divergent primers to detect the expression level of circRNA_1193.

**Fig 5:**
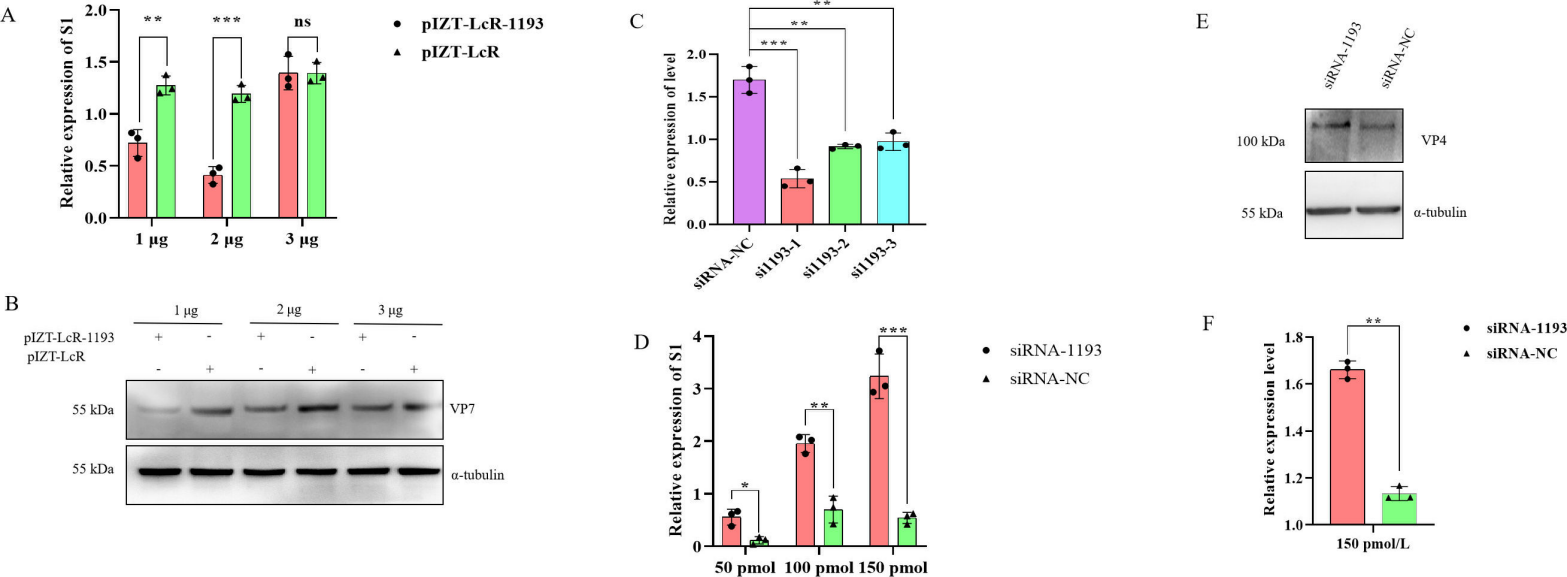
Functional analysis of circRNA_1193 in BmCPV infection. (A) Effect of circRNA_1193 overexpression on BmCPV viral S1 expression. Real-time PCR analysis of BmCPV viral S1 expression in BmN cells overexpressing circRNA_1193 compared with that in cells transfected with control RNA. (B) Effect of circRNA_1193 overexpression on BmCPV viral protein VP7 expression. Western blotting analysis of BmCPV viral protein VP7 expression in BmN cells overexpressing circRNA_1193 compared with that in cells transfected with control RNA. The results revealed a significant reduction in viral replication in samples with circRNA_1193 overexpression (*n* = 3, *P* < 0.01, Student’s *t*-test). (C) The silencing efficiency of the designed small interfering RNAs (siRNAs) was determined via real-time PCR. (D) Effect of circRNA_1193 knockdown on the BmCPV viral load. Real-time PCR analysis of the BmCPV viral load (S1 expression) in BmN cells following the knockdown of circRNA_1193 via specific siRNAs. (E) Effect of circRNA_1193 knockdown on BmCPV viral protein VP4 expression. Western blotting analysis of BmCPV viral protein VP4 expression in BmN cells following the knockdown of circRNA_1193 via specific siRNAs. (F) Densitometric analysis of the VP4 protein bands shown in the panel.

To investigate the role of circRNA_1193 in BmCPV infection, we employed a dual approach: transient overexpression and targeted knockdown. First, we constructed a transient expression vector, pIZT-LcR-circRNA_1193, to overexpress circRNA_1193 in silkworm cells (Fig. 4A through D). The overexpression of circRNA_1193 significantly reduced BmCPV replication, as evidenced by decreased viral gene and protein expression levels, as measured by real-time PCR and Western blotting (Fig. 5A and B). These results suggest that circRNA_1193 may have antiviral activity, potentially by modulating the host immune response or directly interfering with viral replication.

To further investigate the role of circRNA_1193 in BmCPV infection, we performed a knockdown study using specific small interfering RNAs (siRNAs) targeting the junction site of circRNA_1193. This approach aimed to elucidate the consequences of circRNA_1193 loss on viral replication dynamics. Successful knockdown of circRNA_1193 was confirmed by real-time PCR (Fig. 5C), which revealed significant increases in the expression levels of viral genes (Fig. 5D) and proteins (VP4) (Fig. 5E and F) in the siRNA-treated cells. These findings highlight the potential mechanisms by which circRNA_1193 may confer resistance against BmCPV, either through direct interference with viral processes or by enhancing the host immune response.

### CircRNA_1193 does not inhibit BmCPV infection through regulation of its parental gene (*mAAP*)

**Fig 6:**
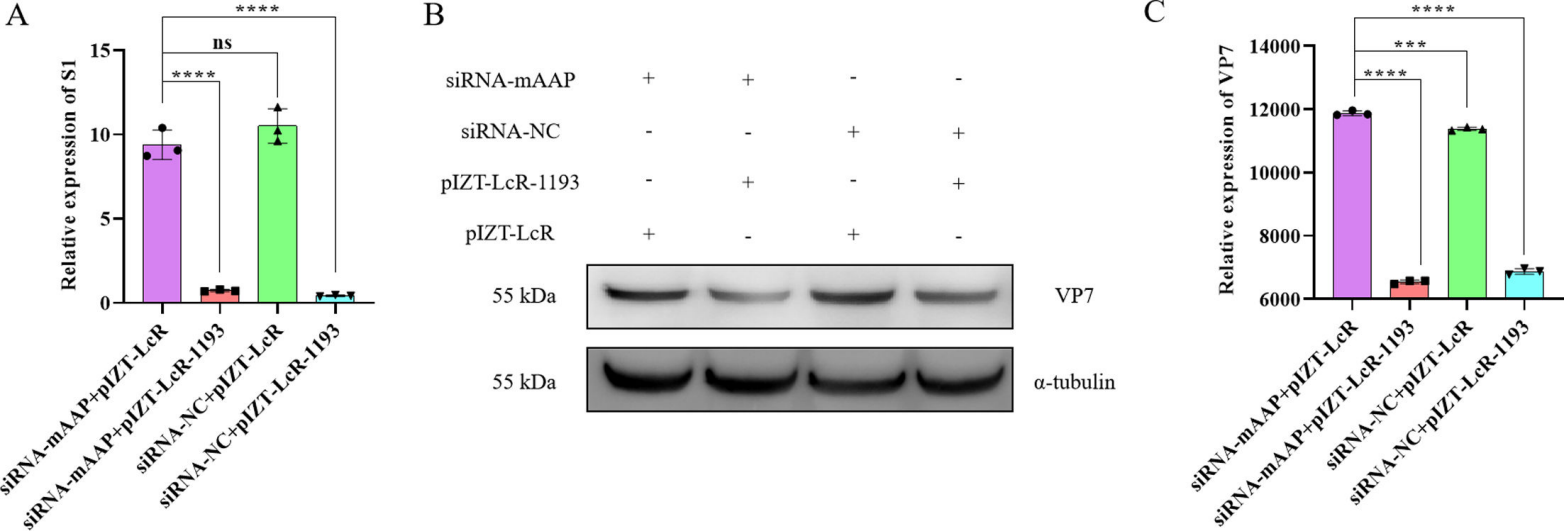
Knockdown of the circRNA_1193 parental gene (*mAAP*) does not affect BmCPV replication levels. (A) Effect of *mAAP* knockdown on BmCPV S1 expression. BmN cells were transfected with 50 pmol of mAAP-specific siRNA or a siRNA-NC and infected with BmCPV 48 h later. The expression level of the BmCPV S1 was measured via real-time PCR. (B) Effect of *mAAP* knockdown on BmCPV VP7 protein expression. BmN cells were transfected with 50 pmol of mAAP-specific siRNA or siRNA-NC and infected with BmCPV 48 h later. The cells were harvested 48 h post-infection, and the expression of the viral protein VP7 was analyzed via Western blotting. α-Tubulin served as a loading control. (C) Densitometric analysis of the VP7 protein bands shown in the panel.

To investigate whether circRNA_1193 modulates BmCPV infection through regulation of its parental gene (*mAAP*), we performed a knockdown experiment. BmN cells were transfected with 50 pmol of mAAP-specific siRNA and pIZT-LcR-circRNA_1193, followed by infection with BmCPV. Forty-eight hours post-infection, total protein and RNA were extracted for Western blotting and real-time PCR analysis. Compared with those in cells transfected with a nontargeting siRNA control (siRNA-NC), there was no significant change in the transcription levels of the BmCPV S1 (Fig. 6A) or in the expression of the viral VP7 protein (Fig. 6B and C). These findings indicate that circRNA_1193 does not inhibit BmCPV infection through the regulation of its parental gene.

### A protein translated from ectopically expressed circRNA_1193

**Fig 7:**
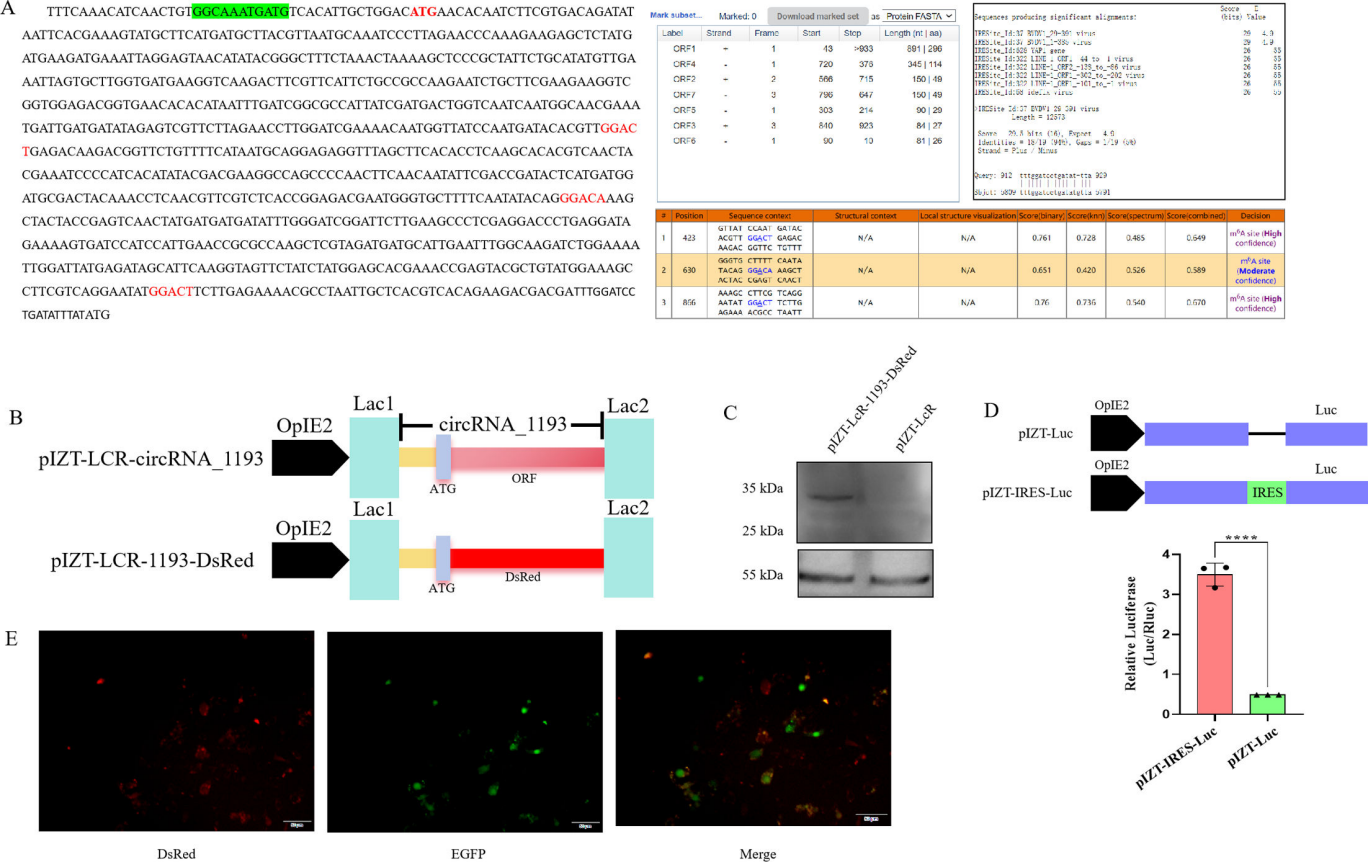
Confirmation of protein translation from ectopically expressed circRNA_1193. (A) Bioinformatics analysis identified potential ORFs, m6A sites, and IRES elements within circRNA_1193. (B) Schematic representation of the pIZT-LcR-circRNA_1193-DsRed expression vector. The ORF sequence in the circRNA_1193 expression vector pIZT-LcR-circRNA_1193 was replaced with the DsRed gene to construct the expression vector pIZT-LcR-circRNA_1193-DsRed. (C) Western blotting analysis of DsRed protein expression in pIZT-LcR-circRNA_1193-DsRed-transfected BmN cells. A total of 1 × 10^5^ BmN cells were inoculated in cell culture dishes and transfected with 2 µg of pIZT-LcR-circRNA_1193-DsRed. The pIZT-LcR plasmid served as a negative control. After 48 h, the proteins were extracted from the cells and analyzed via Western blotting. The primary antibodies used were a DsRed mouse antibody (1:1,000 dilution) and an α-tubulin mouse antibody (1:2,000 dilution), and the secondary antibody used was goat anti-mouse IgG labeled with horseradish peroxidase (HRP) (1:5,000 dilution). The amount of protein loaded per lane was 30 µg. (D) Luciferase activity assays. The predicted IRES-like sequence (GGCAAATGATG) was inserted upstream of the *Luc* gene in the pIZT-V5/His vector to create the pIZT-IRES-Luc construct. The empty pIZT-V5/His vector containing only the *Luc* gene served as the control. BmN cells were transfected with either the pIZT-IRES-Luc construct or the control pIZT-V5/His-Luc vector. Luciferase activity was measured 48 h post-transfection. (E) Fluorescence microscopy image of DsRed expression in pIZT-LcR-circRNA_1193-DsRed-transfected BmN cells.

To investigate the translational potential of circRNA_1193, we performed a series of molecular biology experiments. Bioinformatics analysis revealed a potential rolling circle open reading frame (ORF), three potential m6A methylation sites, and three internal ribosome entry sites (IRESs) elements within circRNA_1193 (Fig. 7A), suggesting the possibility of noncanonical translation. To experimentally confirm the translation, we selected the largest ORF (933 nt/311 aa) and constructed a plasmid (pIZT-LcR-circRNA_1193-DsRed) in which the predicted ORF was replaced with the *DsRed* gene (Fig. 7B). Western blotting analysis also confirmed the expression of a protein band corresponding to the expected size of the DsRed protein (Fig. 7C). To further elucidate the translation mechanism, it was found that the predicted IRES-like sequence (GGCAAATGATG) could significantly enhance the activity of the *Luc* reporter gene (Fig. 7D). Transfection of this construct into BmN cells resulted in the detection of red fluorescence under an inverted fluorescence microscope 48 h post-transfection (Fig. 7E), providing evidence for successful translation from the circRNA_1193 construct.

### CircRNA_1193-dependent virus resistance is mediated by its encoded protein

**Fig 8:**
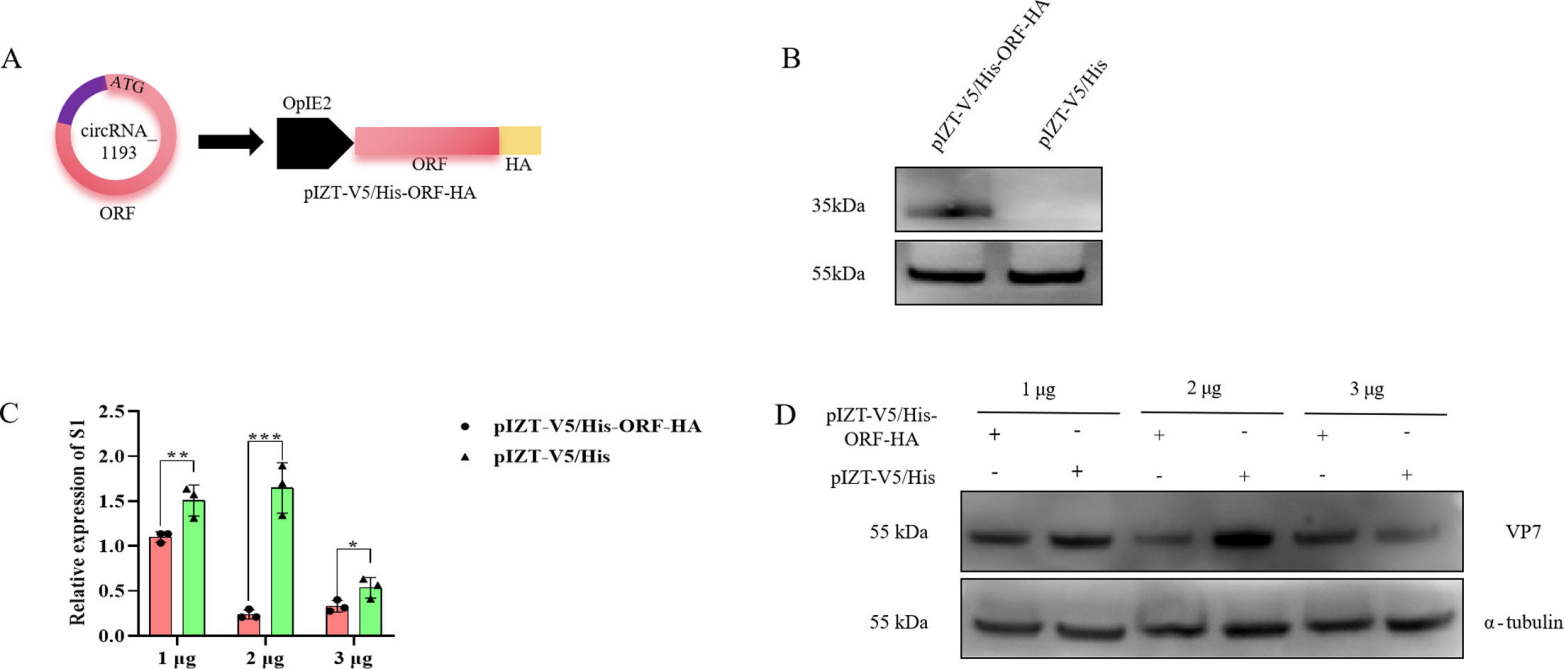
The ORFs encoded by circRNA_1193 inhibit BmCPV infection. (A) Schematic representation of the pIZT-V5/His-ORF-HA plasmid construction. (B) Western blotting analysis of ORF expression in pIZT-V5/His-ORF-HA-transfected BmN cells. (C) Effect of pIZT-V5/His-ORF-HA transfection on BmCPV viral S1 gene expression. A total of 1 × 10^5^ BmN cells were transfected with 1, 2, or 3 µg of the pIZT-V5/His-ORF-HA plasmid and infected with BmCPV (multiplicity of infection [MOI] = 3) 48 h later. The cells were harvested 48 h post-infection, total RNA was extracted, and the expression level of the viral S1 gene was detected via real-time PCR. *RP49* served as the reference gene. (D) Effect of pIZT-V5/His-ORF-HA transfection on BmCPV viral protein VP7 expression. A total of 1 × 10^5^ BmN cells were transfected with 1, 2, or 3 µg of the pIZT-V5/His-ORF-HA plasmid and infected with BmCPV (MOI = 3) 48 h later. The cells were harvested 48 h post-infection and analyzed for VP7 protein expression via Western blotting. The primary antibody used was mouse anti-VP7 (1:2,000), the secondary antibody used was HRP-labeled goat anti-mouse IgG (1:500), and α-tubulin served as the internal control. BmN cells transfected with 1, 2, or 3 µg of pIZT plasmid were used as controls.

To investigate the role of the protein (temporarily named VSP35) encoded by circRNA_1193, we constructed a recombinant expression plasmid, pIZT-V5/His-ORF-HA, containing the circRNA_1193 ORF sequence fused with a hemagglutnin (HA) tag at the C-terminus (Fig. 8A). Transfection of this plasmid into BmN cells resulted in the detection of positive HA signal bands via Western blotting (Fig. 8B), confirming the successful expression of the ORF-HA fusion protein. To assess the effect of ORF expression on BmCPV replication, BmN cells were transfected with increasing doses (1 µg, 2 µg, or 3 µg) of either the pIZT-V5/His-ORF-HA plasmid or an empty vector control (pIZT-V5/His). Forty-eight hours post-transfection, the cells were infected with BmCPV. At 48 h post-infection, total protein and RNA were extracted for analysis. Western blotting was used to detect BmCPV VP7 protein expression (Fig. 8C), while real-time PCR was used to measure BmCPV S1 expression (Fig. 8D). In addition, the expression levels of VP7 (Fig. 8E) and BmCPV S1 (Fig. 8F) were inhibited in the pIZT-V5/His-ORF-HA plasmid-transfected BmN cells after different durations (24, 48, and 96 h) of BmCPV infection. The results demonstrated that ORF expression significantly inhibited BmCPV replication in a dose-dependent manner. These findings provide strong evidence that the protein encoded by circRNA_1193 plays a crucial role in mediating antiviral defense against BmCPV.

### CircRNA_1193-mediated inhibition of BmCPV proliferation involves protein

**Fig 9:**
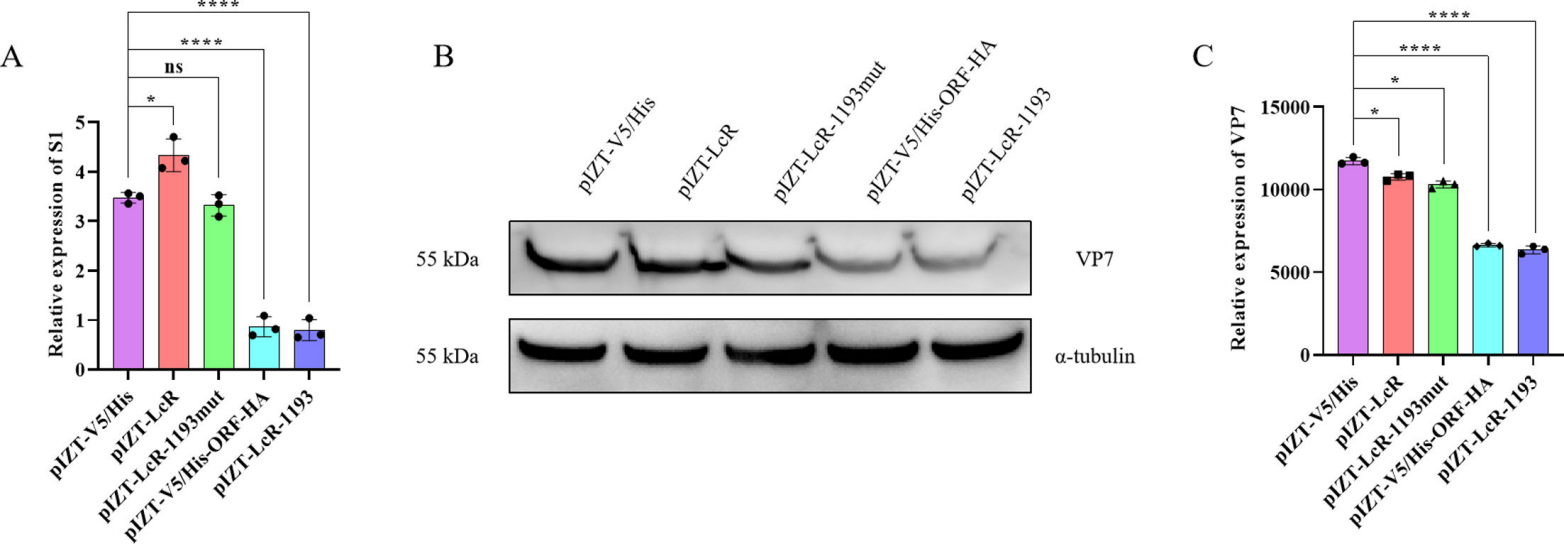
The antiviral activity of circRNA_1193 is mediated by its protein product. (A) Loss of antiviral activity upon mutation of the start codon within circRNA_1193. Real-time PCR analysis of the BmCPV viral load in BmN cells transfected with a mutant circRNA_1193 construct (pIZT-LcR-circRNA_1193mut) lacking a functional start codon. The samples lacking functional circRNA_1193 recovered from BmCPV replication (*n* = 3, *P* < 0.05, Student’s *t*-test). (B) Western blotting analysis of protein expression from circRNA_1193 in silkworm tissues. The presence of a specific band in the overexpressed tissues confirmed successful translation. (C) Densitometric analysis of the Western blotting bands shown in the panel.

To determine whether the antiviral activity of circRNA_1193 is mediated by its encoded protein VSP35, we generated a mutant expression vector, pIZT-LcR-circRNA_1193mut, in which the initiation codon (ATG) was mutated to a stop codon (TAA). This mutation prevents translation of the circRNA_1193 ORF. Both the wild-type (pIZT-LcR-circRNA_1193) and mutant (pIZT-LcR-circRNA_1193mut) plasmids were transfected into BmN cells, which were subsequently infected with BmCPV. Compared with control cells, 48 h post-infection, cells transfected with the wild-type construct exhibited significantly reduced viral replication compared to control cells, confirming the antiviral activity of circRNA_1193. In contrast, cells transfected with the pIZT-LcR-circRNA_1193mut construct, which lacks functional protein expression due to the initiation codon mutation, presented significantly increased levels of viral replication (Fig. 9A). Real-time PCR analysis of BmCPV S1 expression revealed viral loads comparable to control levels in cells transfected with the mutant construct, further supporting the dependence of antiviral activity on the presence of the encoded protein. Western blotting analysis confirmed the successful expression of the encoded protein from the wild-type construct and overexpression vector, which significantly inhibited the expression of VP7, whereas the inhibition of VP7 expression was abolished in the BmN cells transfected with the mutant construct (Fig. 9B and C), confirming that the initiation codon mutation effectively abolished protein translation. These results compellingly demonstrate that circRNA_1193-mediated antiviral activity is indeed mediated through the protein it encodes, highlighting the diverse roles of circular RNAs beyond their regulatory functions.

### Flanking short repeat sequences is sufficient for circRNA_1193 production

**Fig 10:**
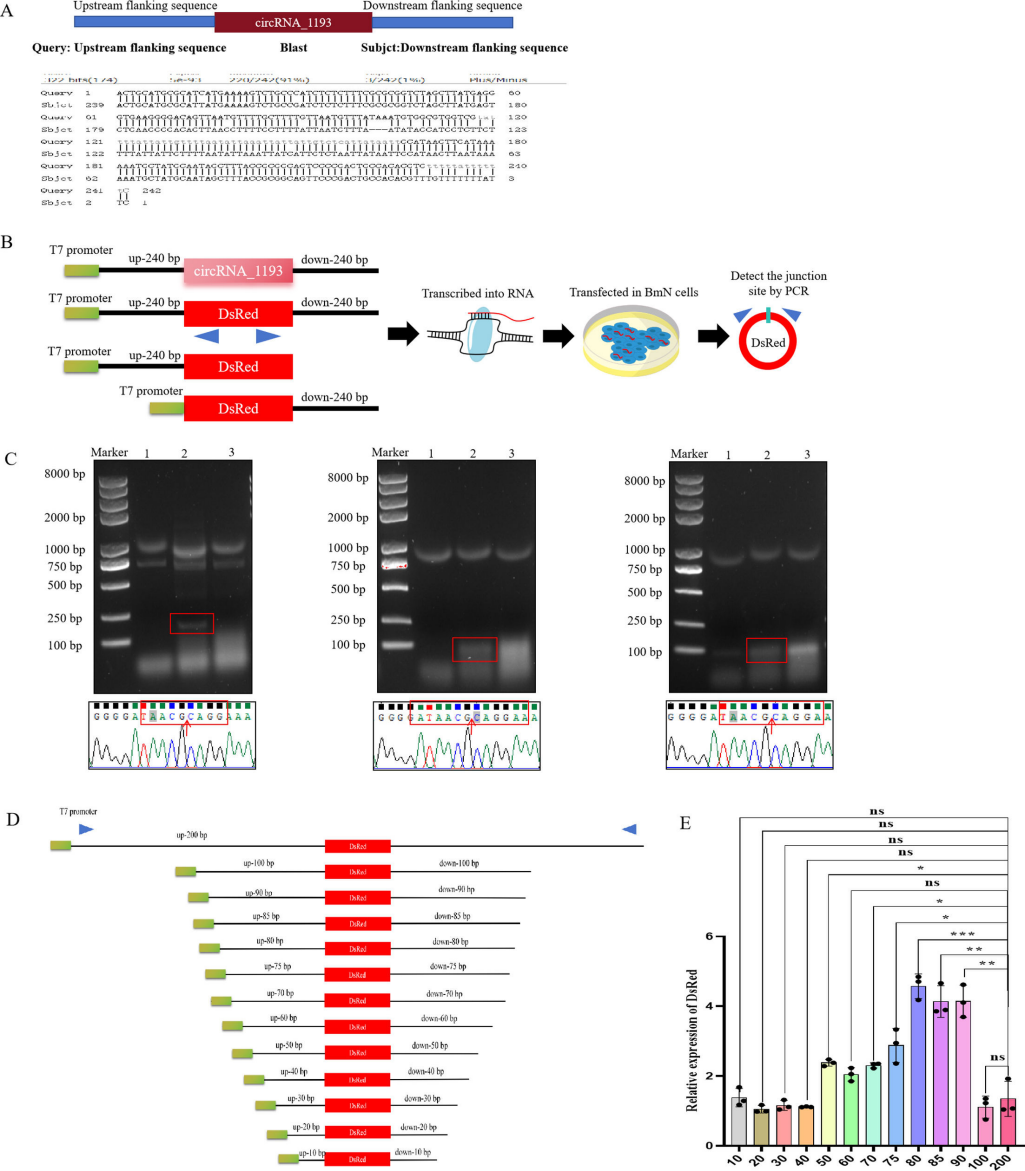
Short repeat sequences are sufficient for circRNA_1193 production. (A) Predicted flanking short repeat sequences for circRNA_1193 formation. (B) Schematic diagram of the pIZT-V5/His-circRNA_1193-arm-DsRed plasmid and cell transfection. Two highly repetitive sequences, approximately 240 bp in length, were selected from the 5´ and 3´ regions of circRNA_1193 as the left and right arm sequences, respectively. A DsRed sequence was inserted between the left and right arm sequences. (C) Influence of the short repeat sequence on circRNA_1193 formation. Different RNA fragments were synthesized *in vitro* via T7 polymerase and transfected into BmN cells, CIK cells, and L929 cells. Total RNA was extracted 48 h later, and divergent primers were used to amplify the junction site. The amplified products were analyzed by electrophoresis and Sanger sequencing. The red arrow indicates the junction site. BmN cells (lane 1): transfected with the 240 bp upstream sequence (up-240 bp). CIK cells (lane 2): transfected with both the upstream (up-240 bp) and downstream (down-240 bp) sequences. L929 cells (lane 3): transfected with the downstream 240 bp sequence (down-240 bp). (D) A series of plasmids containing these sequences with varying sizes (200, 100, 90, 85, 80, 75, 70, 60, 50, 40, 30, 20, and 10 bp) as the left and right arms, with a *DsRed* gene inserted between them, were constructed. Different RNA fragments were synthesized *in vitro* via T7 polymerase and transfected into BmN cells. (E) The effects of flanking sequences on circRNA formation efficiency were detected via real-time PCR.

To investigate the mechanism of circRNA_1193 formation, we sought to determine whether short repeat sequences play a critical role in its circularization. We focused on sequences flanking exons 2 and 9 of the *mAAP* gene, which were predicted to facilitate the circularization process. Sequence analysis of the *mAAP* gene revealed a 240 bp region containing two highly repetitive sequences (Fig. 10A). To determine whether circRNA biogenesis is specific to cell species or dependent on sequence elements, we constructed a series of plasmids. Each plasmid contained two natural flanking short repeat sequences (240 bp), with either the right or left arm deleted (Fig. 10B). Different RNA fragments were synthesized *in vitro* via T7 polymerase and transfected into BmN cells, CIK cells, and L929 cells. Total RNA was extracted 48 h post-transfection, and divergent primers were used to amplify the junction site. Electrophoresis and Sanger sequencing revealed that only the RNA fragments containing both natural flanking short repeat sequences could form a detectable circRNA junction site, which was specifically observed in BmN cells (Fig. 10C). This finding suggests that while the presence of specific sequence elements is crucial for circRNA biogenesis, the process may also exhibit some cell type specificity, at least in the case of BmN cells.

To test the functionality of these repeats, we constructed a series of plasmids containing these sequences with varying sizes (200, 100, 90, 80, 70, 60, 50, 40, 30, 20, and 10 bp) as the left and right arms, with a red fluorescence protein (*DsRed*) gene inserted between them (Fig. 10D). These constructs were designed to produce circRNAs containing the *DsRed* gene sequence. Various segments of the RNA constructs were synthesized via T7 RNA polymerase and transfected into BmN cells. The efficiency of flanking sequences on circRNA formation was detected with real-time PCR, and the results revealed that the short repeat sequences between 70 and 80 bp may play a crucial role in the circularization process of circRNA_1193 (Fig. 10E), validating the hypothesis that these short repeat sequences are sufficient for circRNA formation. Our findings demonstrate that short repeat sequences flanking the *mAAP* gene are essential for the production of circRNA_1193.

## DISCUSSION

In this study, we have elucidated the role of circRNA_1193 in the context of BmCPV infection, revealing its potential as a novel protein-coding circular RNA that exerts antiviral effects. Our findings demonstrated that circRNA_1193 is not only expressed in silkworm tissues but also modulated in response to viral infection, indicating its involvement in the silkworm defense mechanisms against BmCPV.

CircRNAs have gained attention for their diverse functions, particularly in regulating gene expression and influencing viral infections (6, 19). The unique circular structure of circRNA endows it with stability and the ability to engage with specific microRNAs or proteins, thereby modulating various biological pathways (11, 13). Our results align with this notion, as the overexpression of circRNA_1193 resulted in a significant reduction in BmCPV replication, whereas its knockdown had the opposite effect. These findings suggest that circRNA_1193 may serve as an important negative regulator of viral replication in silkworms.

The coding potential of circRNAs has been an intriguing topic of recent research, with several studies reporting that circRNAs can indeed produce functional proteins (9, 15, 16, 20). Our work further supports this concept, as the experimental mutation of the start codon within circRNA_1193 abolished its inhibitory effect on viral replication, emphasizing the necessity of its translated protein in mediating the antiviral response. These findings suggest that circRNA_1193 may function analogously to traditional antiviral proteins, contributing to the host’s defense against viral pathogens.

Furthermore, the interaction between circRNA_1193 and miRNAs is another significant aspect of our findings. The ability of circRNAs to function as miRNA sponges has been well documented (10, 11, 16, 21). By sequestering miRNAs, circRNA_1193 may indirectly increase the expression of target genes that contribute to the antiviral response in silkworms. This regulatory network provides a nuanced layer of control over gene expression, allowing the host to tune its response to viral infections finely. In addition to their roles in gene regulation, circRNAs can also modulate host immune responses. Several studies have shown that circRNAs can influence the signaling pathways involved in innate immunity. For example, circMORC3 in *Miichthys miiuy* negatively regulates antiviral immunity by synergizing with the host gene *MORC3* (22). *Drosophila* circZfh1 encodes the CRAV protein, which activates antiviral immunity via the JAK/STAT pathway (9). We also reported that the overexpression of circRNA_1193 in BmN cells significantly increased the expression level of zinc finger 729, whereas the depletion of zinc finger 729 expression level with siRNA could significantly increase the expression level of BmCPV S1, suggesting that circRNA_1193 inhibited viral replication by regulating the miR-277-5p/zinc finger 729 axis (data not shown). Future investigations into the circRNA_1193/miR-277-5p/zinc finger 729 axis could reveal its broader impact on the host’s ability to mount an effective defense against BmCPV.

Moreover, we determined that the formation of circRNA_1193 is closely related to the reverse complementary flanking sequences. In particular, the short repeat sequences between 70 and 80 bp may play a pivotal role in the formation of circRNA_1193. In addition, the biogenesis of circRNA dynamically changes with the truncation of the reverse complementary flanking sequences, suggesting that various factors may bind to reverse complementary sequences to either promote or inhibit the formation of circRNAs. This highlights the intricate mechanisms underlying circRNA biogenesis, which may be influenced by the structural configurations and sequence complementarities of precursor mRNAs (23). Understanding these mechanisms can increase our ability to manipulate circRNA expression for therapeutic purposes, particularly in the context of viral infections.

Our findings indicate that circRNAs may emerge as essential components of antiviral strategies, not only in silkworms but also in other species, owing to their evolutionary conservation and functional versatility. In conclusion, our study demonstrated that circRNA_1193 plays a vital role in the antiviral response of silkworms to BmCPV, providing new insights into the complex interplay between circRNAs and viral pathogens. Further exploration of circRNA dynamics in virus-host interactions may reveal new strategies for enhancing viral resistance in silkworms and other species.

## MATERIALS AND METHODS

### Cell culture

BmN cells obtained from the Molecular Biology Lab of Soochow University were cultured at 26°C in TC-100 medium (Sangon Biotech, Shanghai, China) supplemented with 10% fetal bovine serum.

### Viral infection

BmN cells were seeded in six-well plates at a density of 1 × 10^5^ cells per well and cultured overnight. The cells were then infected with BmCPV. To allow virus particle attachment, the cells were incubated at 4°C for 1 h, followed by incubation at 26°C for 1 h in TC-100 medium supplemented with 10% fetal bovine serum.

### Prediction of the coding potential of circRNA_1193

The potential for protein-coding within the circRNA_1193 sequence was predicted via ORF Finder online software (https://www.ncbi.nlm.nih.gov/orffinder/). Potential m6A methylation modification sites on circRNA_1193 were analyzed via SRAMP (http://www.cuilab.cn/sramp). IRESs were predicted via IRESite (http://www.iresite.org/).

### RT-PCR and Sanger sequencing

The presence of circRNA_1193 was verified via reverse transcription PCR using divergent and convergent primers (Table 1). Total RNA was extracted from BmN cells via RNAisoPlus (Takara, Dalian, China), and linear transcripts were removed by treating total RNA with RNase R (Epicentre). The RNA was then reverse-transcribed into cDNA via random primers. RT-PCR amplification was performed via divergent and convergent primers, and the amplified products were ligated into the pMD-19T vector (Takara, Dalian, China). Sanger sequencing was then performed at Sangon Biotech, Shanghai, China.

**TABLE 1:** The primers and siRNAs used in this study

Name	Forward primer	Reverse primer
circ_9032 divergent primer	GCTGAACGCGTCACAACAA	CCACTGACTTGACTGATATG
circ_3493 divergent primer	GTGTGACCTCGATCGATA	GAAAGCTAGCACACATGCGT
circRNA_1193 divergent primer	GAGAAAACGCCTAATTGCTC	TCTGTCACGAAGATTGTGTT
circ_9582 divergent primer	CAGAAGTACGAACGCATCA	AGTACGACTACACGTTCGAC
circRNA_1193 QC primer	TGGATCCTGATATTTATATGTTTCAAACAT	AATCGTCGTCTTCTG
circRNA_1193 line primer	TTGTTGGCATTTGGGAACC	TCTTCTTTGGGTTCTAAGGGA
circRNA_1193 probe	bio-GGGTACAGCATCTCGGTGTT	
Rp49	ACTCTGATGCTGAGCTGCTG	GACCTGTTTACAGGCCGACA
S1	GGTCTCGACGTGAATACCGA	TCGTCTGCTTCACTAGCACG
siRNA-1	UAUAUGUUUCAAACAUCAACUTT	AGUUGAUGUUUGAAACAUAUATT
siRNA-2	UUUAUAUGUUUCAAACAUCAATT	UUGAUGUUUGAAACAUAUAAATT
siRNA-3	AUAUUUAUAUGUUUCAAACAUTT	AUGUUUGAAACAUAUAAAUAUTT
T7-left	TAATACGACTCACTATAGGGGTACCACTGCATGCGCAT	
T7-right	TAATACGACTCACTATAGATGAGCGAGCTGATTAAGGAGAA	ACCGGTACTGCATGCGCA
T7-left-right	TAATACGACTCACTATAGGGGTACCACTGCATGCGCAT	ACCGGTACTGCATGCGCA
RFP	AAGCTGTACATGGAGGGCAC	TGGGACGTCGTATGGGTACT
277-5P primer	GCCGAGTCGTGCCAGGAGTGC	GCAGGGTCCGAGGATTC
Zinc protein primer	TTCCGTGTCTGTTCTGTCGG	GAAGCCGTTGTGAGTCTGGA
circRNA_1468 primer	ACAAGAAGATATGGTGGCAAAT	GGTCGAGCTGGCTGG
circRNA_2200 primer	ACGTGAAGAGGTATTTG	GCTGATTTATCCATCCATGCGGT
circRNA_4943 primer	GGACCTGGACGCAGC	TTGGCCAGCTTCCTGTTC
circRNA_5322 primer	TTCGTGGGCTTCACGTACA	TTCCCTTTCGAGCGCTTT
circRNA_6596 primer	AGCTAAACTCCAAGTGAAGAAAC	GCGTCTCTTTCATTTGACG
circRNA_5354 primer	GTCCAAGAAACAGTCTCACG	CTAGCCGCGGTCAAAATC
circRNA_3827 primer	GCCGCAGGAGCTCTGCCC	GGCTGCGGCTGGGGCG
circRNA_5663 primer	AACGCCCATCTAGGCATAG	GTTCGTACTTCGCTTTTTCGT
circRNA_5664 primer	GGCAAAATTACGTGAAGAGG	TGATTTATCCATGCGGTCAG
circRNA_4541 primer	ACCACCAGGAGAAGACG	CCAACGGCTGATTTATCCAT
circRNA_1029 primer	CTTACCGCGAAGACAAAAAT	CGTACTTCTGCTTTTTCGTG
circRNA_1750 primer	GCGTTCGTACTTCTGCTTTT	GAACTATCAGTACGACTACACG
circRNA_4499 primer	CAACAGCGCGTTTTCCCTT	GATGCTGATCAACGAGAACAGG
circRNA_3285 primer	GTGGGCTTCACGTACAC	AGCTCGGCTATGGTCTG
circRNA_3092 primer	AGTCCAAGAAACAGTCTCAC	GACTCCTTCCTTGGGGAAAT
circRNA_0325 primer	AATGTGACCCACACTTGAAC	TCATTTGACGTTGTTGTGA
circRNA_3104 primer	ATGCCCATTCGATTTTGACC	CAGTTTTCTTGACAGCATCC
circRNA_4088 primer	GCAGTTCAAGTGTGGGTC	CTTCAGTTTTCTTGACAGCA
circRNA_3184 primer	AGCGCCGTCCAACG	GCGTCCAGGTCCTTGG

### Northern blotting

The presence of circRNA_1193 was confirmed by Northern blotting. Total RNA was extracted from BmN cells via RNAisoPlus (Takara, Dalian, China) and treated with RNase R (Epicentre) to remove linear RNA transcripts. The treated RNA was separated on a 1% agarose-formaldehyde gel and then transferred onto a Hybond-N membrane (GE Healthcare, USA). The membrane was hybridized with a biotin-labeled DNA probe (bio-GGGTACAGCATCTCGGTGTT) designed on the basis of the junction site of circRNA_1193.

### *In situ* hybridization

A total of 1 × 10^5^ BmN cells were cultured in 24-well plates for 24 h, and *in situ* hybridization was performed via a biotin-labeled oligonucleotide probe (bio-GGGTACAGCATCTCGGTGTT) designed on the basis of the junction site of circRNA_1193. A strong red fluorescence signal was detected in the cytoplasm of BmN cells via an inverted fluorescence microscope (Nikon TE2000-E).

### Construction of circRNA expression vectors

The circRNA expression vector pIZT-LcR was constructed as previously described (16). Multiple cloning sites were inserted between introns 1 (128–548 bp) and 2 (1,169–1,707 bp) of the fruit fly *lacZ* gene. The cDNA sequence of circRNA_1193 was inserted into the pIZT-LcR vector via seamless cloning, generating pIZT-LcR-circRNA_1193. The predicted ORF within circRNA_1193 was replaced with the *DsRed* sequence, resulting in pIZT-LcR-circRNA_1193-DsRed. The predicted ORF sequence tagged with HA at the C-terminus was inserted into the *Kpn* I*/Sac* II site of pIZT-V5/His to construct pIZT-V5/His-ORF-HA. A mutated version of the expression vector was designated pIZT-LcR-circRNA_1193mut. In this construct, we specifically mutated the initiation codon from ATG (the typical start codon for protein translation) to TAA (a stop codon). A series of plasmids containing these sequences with varying sizes (240, 200, 100, 90, 80, 70, 60, 50, 40, 30, 20, and 10 bp) as the left and right arms, with a red fluorescence protein (*DsRed*) gene inserted between them, were constructed. The predicted IRES-like sequence (GGCAAATGATG) along with the *Luc* gene was synthesized and inserted into the pIZT-V5/His vector to create pIZT-IRES-Luc. The empty pIZT-V5/His vector containing only the *Luc* gene served as the control vector.

### siRNA

Specific siRNAs (Table 1) targeting the junction site of circRNA_1193 were synthesized by Integrated Biotech Solutions Corporation (Shanghai, China).

### Cell transfection

BmN cells were seeded in six-well plates at a density of 1 × 10^5^ cells per well. Two micrograms of siRNA or other plasmids was mixed with an equal volume of Lipofectamine 2000 reagent (Invitrogen, Frederick, MD, USA) and transfected into BmN cells. Each transfection was performed independently in triplicate.

### Real-time PCR

Total RNA was extracted from BmN cells infected with BmCPV or transfected with plasmids via RNAisoPlus (Takara, Dalian, China). The RNA was reverse-transcribed into cDNA via random primers. The expression levels of silkworm and viral circRNAs and genes were measured via real-time PCR using SYBR Green (Takara, Dalian, China). *RP49* served as the reference gene. The relative expression levels were calculated via the 2^-ΔΔCT^ method (24).

### Western blotting

Total protein was extracted from BmN cells infected with BmCPV or transfected with plasmids. The protein concentration was determined via a BCA protein assay kit (Thermo Scientific). The quantified proteins were subjected to sodium dodecyl sulfate-polyacrylamide gel electrophoresis. The proteins were then transferred from the gel onto a nitrocellulose membrane (Millipore) for immunodetection. The primary antibodies used were anti-VP7 (homemade), anti-HA-tag (Proteintech), anti-DsRed (Proteintech), and anti-α-tubulin (Proteintech). Horseradish peroxidase-labeled goat anti-mouse IgG (Cell Signaling Technology) served as the secondary antibody. The protein expression levels of visible bands detected via Western blotting were quantified with ImageJ (version 1.48).

### Statistical analysis

All experiments were performed independently at least three times with consistent results. The Western blotting images are representative of three independent experiments. The quantitative data are presented as the mean ± SDs (*n* = 3). Statistical significance was assessed via a two-tailed Student’s *t*-test for comparisons between the two groups. Significance levels are defined as follows: n.s., not significant; *P < 0.05; **P < 0.01; ***P < 0.001.
